# Fibromatosis of the accessory breast mimicking a malignant tumor: a rare case report and literature review

**DOI:** 10.3389/fonc.2025.1548521

**Published:** 2025-06-19

**Authors:** Jing Li, Chaoyi Qi, Yixiao Liu, Jiahuan Xu, Lei Cao, Yanyan Hu, Jian Wang

**Affiliations:** ^1^ Taian Hospital of Traditional Chinese Medicine, Shandong University of Traditional Chinese Medicine Affiliated Hospital, Taian, Shandong, China; ^2^ Affiliated Taian City Central Hospital of Qingdao University, Taian, Shandong, China

**Keywords:** extra-abdominal fibromatosis, case report, accessory breast, surgery, histopathology

## Abstract

Fibromatosis is a rare neoplasm characterized by fibroblastic and myofibroblastic proliferation. Fibromatosis that occurred in the accessory breast has never been reported in the literature worldwide. We present a case of extra-abdominal fibromatosis of the accessory breast occurring in a 36-year-old Chinese female patient. The tumor was hard with unclear boundaries and adhered to surrounding tissues (6 × 4 × 3 cm). Despite that malignancy was initially suspected based on clinical, ultrasound, and magnetic resonance imaging (MRI) results, histopathology and immunohistochemistry showed an unexpected outcome. At the 2-year postoperative follow-up, there was no recurrence; the prognosis was explained to the patient. This case emphasizes the importance of clinical suspicion and histopathological evaluation and the need to raise awareness to promote early diagnosis and appropriate management of fibromatosis. We also present a literature review of varied presentations and treatment options for extra-abdominal fibromatosis.

## Highlights

- Fibromatosis of the accessory breast has never been reported in the literature.
- Ultrasound examination and MRI raised suspicion of malignancy.
- The patient has been followed up with no new complaints in the last 2 years.

## 1 Introduction

Desmoid-type fibromatosis (DF) is also known as aggressive fibromatosis or desmoid tumor (1). The World Health Organization defines DF as a clonal fibroblast proliferation that occurs in the fascia, tendon membrane, or deep soft tissue, characterized by invasive growth and a tendency toward local recurrence, but without the ability to metastasize (2). It often infiltrates and grows in adjacent muscles or adipose tissue, and sometimes, the tumor can also invade nearby important structures and solid organs. If not completely removed during surgery, it is highly prone to recurrence. Therefore, we considered DF a low-grade malignant tumor (3). DF accounts for 3.5% of all fibrotic tumors and 0.03% of all tumors (4). DF can be divided into abdominal fibromatosis, extra-abdominal fibromatosis, and intra-abdominal and mesenteric fibromatosis based on the location of occurrence. Extra-abdominal fibromatosis (EAF) is an extraordinarily rare disease, with a peak incidence in the age range of 30–40 years, affecting two to four patients per 1 million in a population annually. It mainly occurs in the upper limbs (shoulders and arms), chest wall, back, thighs, head, and neck, with a few cases possibly related to silicone implants (5). EAF is multi-focal in approximately 10% of cases and may occur at the site of previous surgery or trauma (6). DF affects women at a disproportionately higher rate compared to men (7). In this report, we describe a case of an EAF of the accessory breast. Under ultrasound and MRI examination, it may be easily misdiagnosed because of its tendency toward malignant tumor morphology. Therefore, we describe our ultrasound, MRI, and pathological observations and review the literature in order to improve our understanding of the disease, avoid misdiagnosis, and provide evidence for its clinical treatment and prognosis.

## 2 Case report

**Figure 1:**
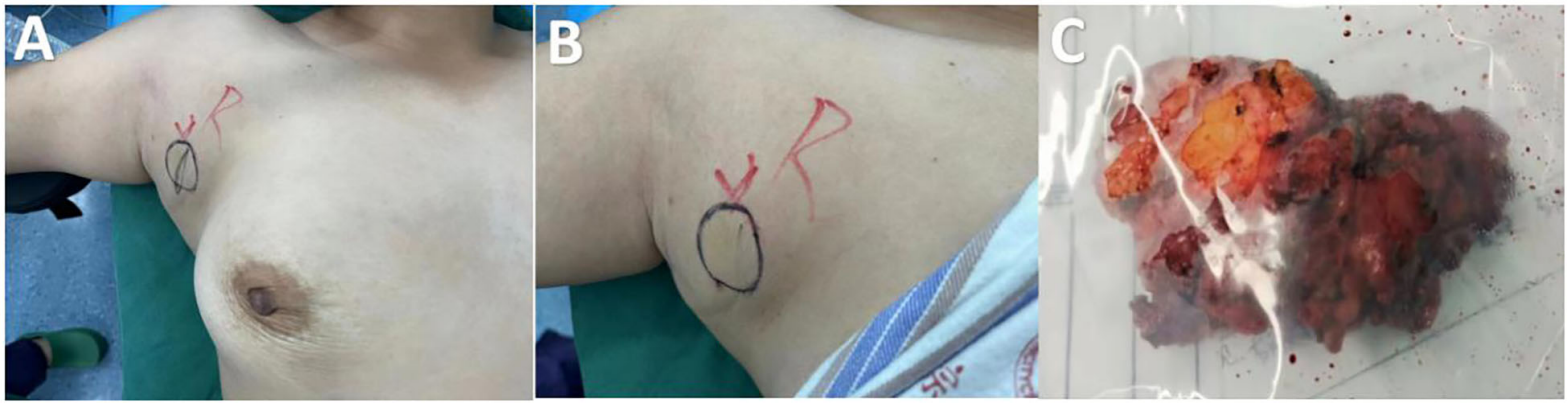
Frontal view of the right axillary mass of preoperative view **(A, B)**. Post-excision of mass **(C)**.

A 36-year-old woman was admitted to the Department of Breast Surgery, Tai’an City Central Hospital, Qingdao University (Tai’an, Shandong Province, China) because a mass in the right accessory breast was accidentally discovered 4 days earlier (**Figures 1A, B**). There was no redness or swelling around the mass, occasional stinging sensation, no nipple discharge or bleeding, and no nipple retraction or deviation. Clinical examination revealed a tough mass approximately 3 × 3 cm in size in the right accessory breast, with unclear boundaries, irregular shape, poor mobility, and no involvement of the skin. There was no obvious mass in either breast. The patient had a history of good physical health and had not suffered from any other illnesses. She underwent a cesarean section in 2010 and 2015. Her father passed away due to liver cancer, but her mother was healthy. The patient had no history of prior DF, no history of major trauma, and no significant family history.

**Figure 2:**
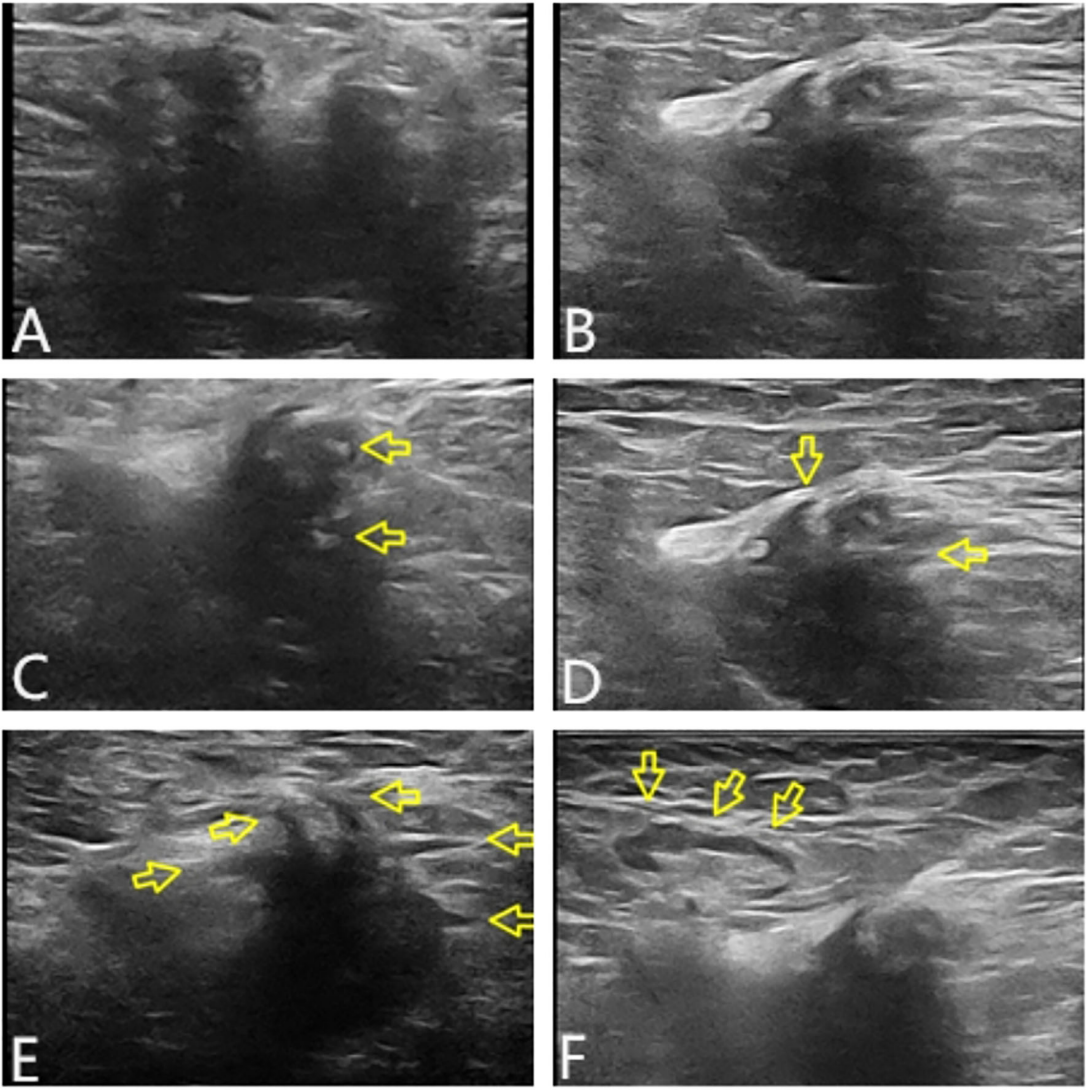
Tumor characteristics on UT. In transverse section, the boundary was unclear, the shape was irregular, and lateral acoustic shadows could be seen around the periphery **(A)**. In longitudinal section, some edges were still smooth, and the posterior echo was enhanced **(B)**. The internal echo of the mass was heterogeneous, with patchy strong echoes visible (arrows in panel **C**). The edge of the lump was angled and showed serrated changes (arrows in panel **D**). Partial section showed crab foot sign (arrows in panel **E**). Lymph node echoes could be seen around, with clear boundaries and regular morphology. The boundary between the cortex and medulla was clear, and the lymph node hilum was centered (arrows in panel **F**). UT, ultrasound.

Ultrasound examination showed a heterogeneous hypoechoic mass in the right axilla, with unclear boundaries and irregular morphology. Some sections showed a crab foot sign. Breast glandular tissue and adipose tissue were seen around the lump, and the boundary between the lump and the main breast was still clear. Lymph node echoes were seen around the mass, with clear boundaries and regular morphology. The corticomedullary boundary of the lymph nodes was clear, and the lymph node hilum was centered. The ultrasound finding raised suspicion of malignancy in the accessory breast and characterized it according to the Breast Imaging Reporting and Data System (BI-RADS) as BI-RADS 4b (**Figure 2**).

**Figure 3:**
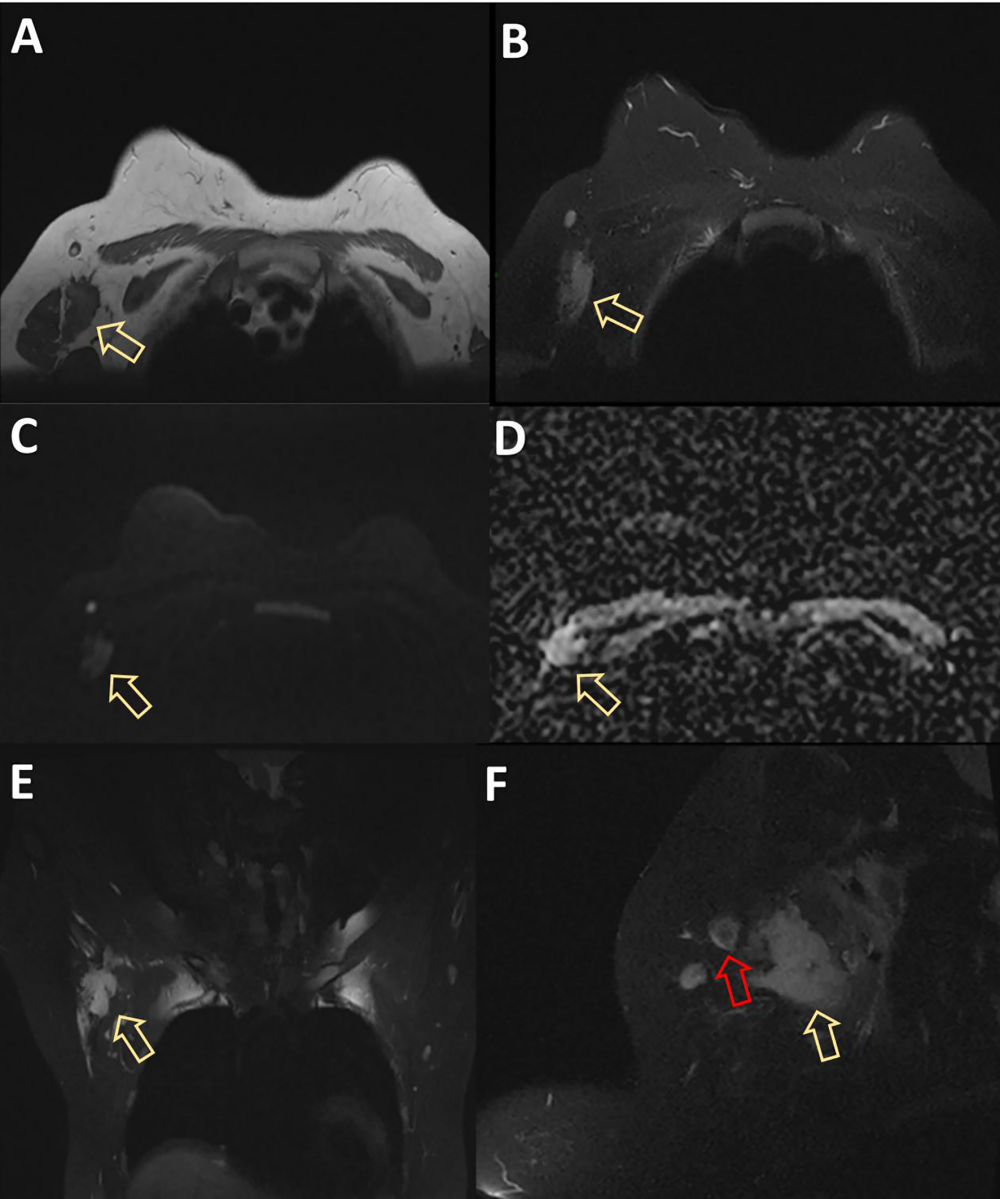
MRI displayed patchy abnormal signal foci in the right axillary area, showing low T1 and high T2 signals (arrows in panels **A**, **B**), without significant diffusion limitation on DWI **(C)**. ADC showed no significant decrease **(D)**. The coronal and sagittal views showed clear boundaries between the mass and the right breast (yellow arrows in panels **E**, **F**), and small lymph nodes with a diameter of approximately 1 cm could be seen at the edge of the mass (red arrow in panel **F**). DWI, diffusion-weighted imaging; ADC, apparent diffusion coefficient.

**Figure 4:**
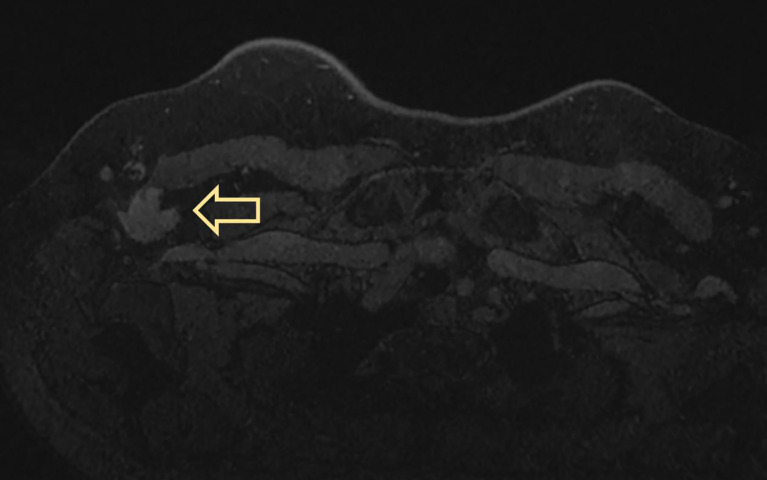
The enhanced scan of the mass shows moderate enhancement.

**Figure 5:**
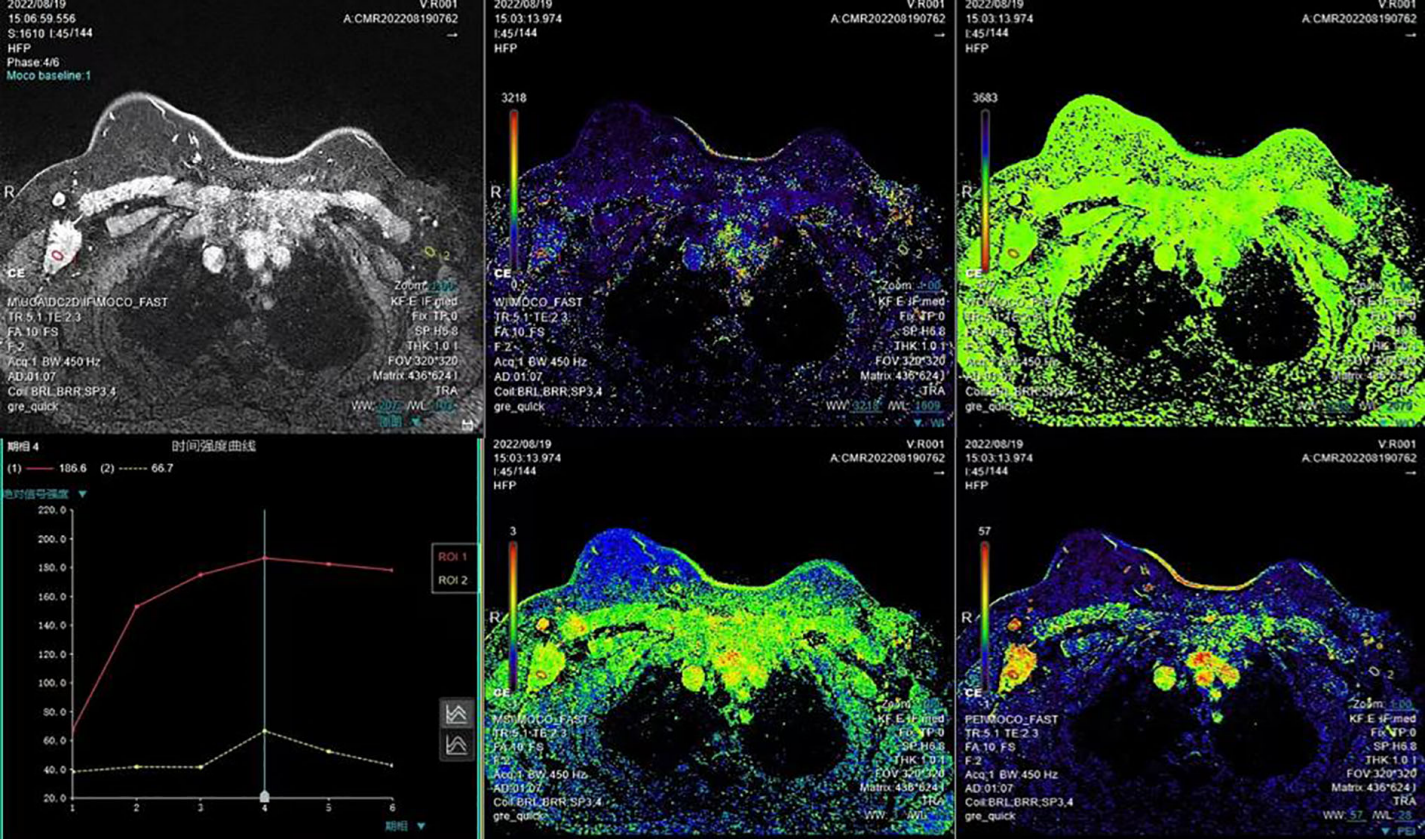
Selected a time resolution of 120 seconds, calculated the early enhancement rate at 2 minutes after injection, and selected the curve change trend after the third minute as the basis for contour judgment. Dynamic enhancement led to an increase in signal intensity of early lesions, with an increase or decrease of less than 10% in signal intensity in the middle and later stages. Dynamic Contrast-Enhanced (DCE)-TIC curve showed a type II, plateau type. TIC, time–signal intensity curve.

MRI displayed a patchy abnormal signal focus in the right axillary area, diffusion-weighted imaging (DWI) detected no significant diffusion limitation, apparent diffusion coefficient (ADC) showed no significant decrease, and small lymph nodes could be seen at the edge of the mass (**Figure 3**). The enhanced scan of the mass showed moderate enhancement (**Figure 4**). The time–signal intensity curve (TIC) showed a type II, plateau type (**Figure 5**). MRI raised suspicion of malignancy in the accessory breast and characterized it as BI-RADS 4b.

**Figure 6:**
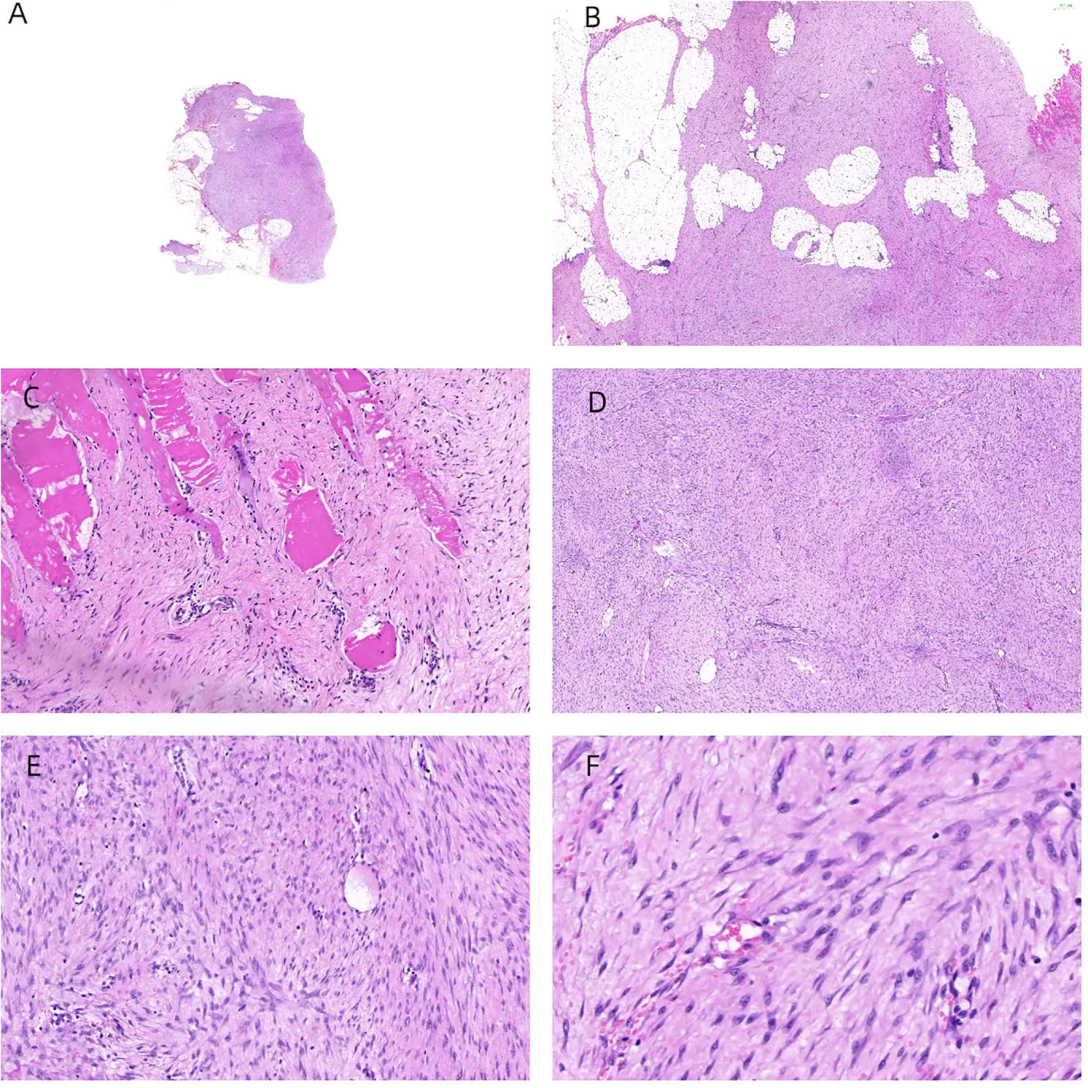
H&E staining of the samples. The tumor was located within the accessory breast tissue of the axilla, with unclear boundaries. Tissue sections showed star-shaped features and invasive growth patterns **(A, B)**. The tumor infiltrated and grew within striated muscle tissue **(C)**. Under a low-magnification microscope, spindle-shaped cells were arranged in bundles and bands in a staggered pattern **(D)**. Fibroblasts or myofibroblasts were arranged in bundles or bands, with scattered small blood vessels visible (×100 magnification) **(E)**. Tumor cells were spindle-shaped, with slightly alkaline cytoplasm, oval-shaped nuclei, pale nuclear chromatin, and small nucleoli (×200 magnification) **(F)**. Mitosis was rare—1/10 HPF.

**Figure 7:**
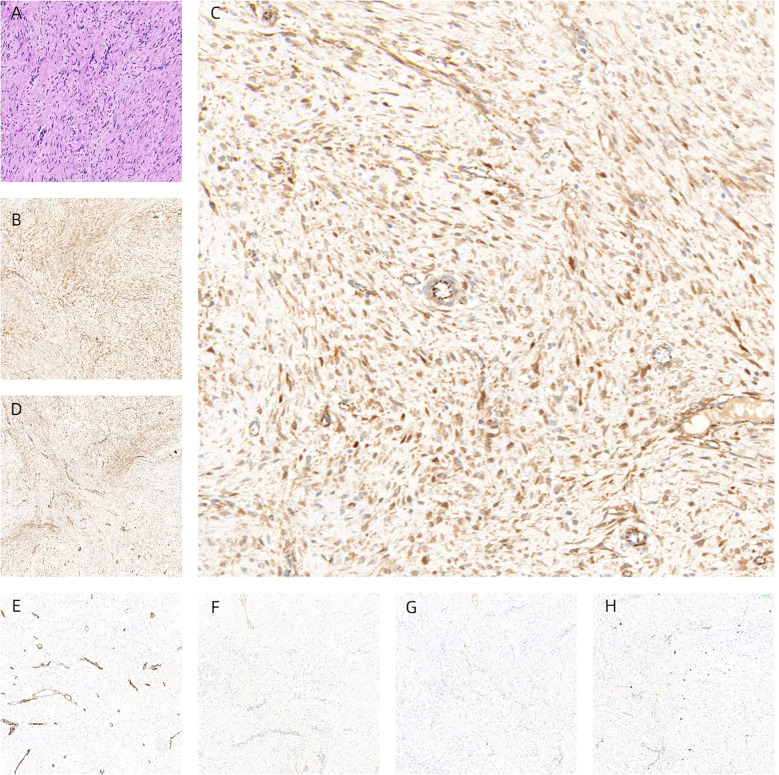
Tumors were composed of gently shaped fibroblasts, and myofibroblasts were arranged in bundles **(A)**. Immunohistochemically, the tumor cells were positive for β-catenin **(B, C)** and smooth muscle actin **(D)**. CD34 **(E)**, p63 **(F)**, and CK **(G)** had no immune reactivity. Ki67 immunostaining showed a cell positivity index of approximately 5% **(H)**, interpreted as desmoid fibromatosis.

Following the clinical assessment by the Department of Breast Surgery, surgical treatment was planned immediately. The operation was performed under general anesthesia in the horizontal supine position. An approximately 6 × 4 × 3 cm mass was found in the accessory breast of the right axilla, which was hard with unclear boundaries and adhered to surrounding tissues (**Figure 1C**). The tumor was completely removed and sent for rapid pathology. Under a low-magnification microscope, it was found that spindle-shaped cells were arranged in bundles and bands in a staggered pattern. Fibroblasts or myofibroblasts were arranged in bundles or bands, with scattered small blood vessels visible. Tumor cells were spindle-shaped, with slightly alkaline cytoplasm, oval-shaped nuclei, pale nuclear chromatin, and small nucleoli. Mitosis was rare—1/10 HPF (**Figure 6**). Immunohistochemically, the tumor cells were positive for β-catenin and smooth muscle actin. CD34, p63, and Cytokeratin (CK) had no immune reactivity (**Figure 7**).

Based on these findings, the tumor was finally diagnosed as EAF, the immediate post-op period was uneventful, and the patient has been on regular follow-up with no new complaints about her symptoms in the last 2 years.

## 3 Discussion

We report an extremely rare case of fibromatosis occurring in the accessory breast. The patient had no history of breast surgery or trauma. She came to our hospital for a visit due to the accidental discovery of a mass in the axilla. We summarize the ultrasound (UT) and MRI findings and the histopathological manifestations of this unusual tumor.

DF often presents as painless isolated masses with hard texture, poor mobility, and unclear boundaries, and it may adhere to deep muscles. Therefore, symptoms such as pressure on adjacent tissues, joint stiffness, or gastrointestinal discomfort may also emerge as a result of tumor growth (8). Lesions in the head and neck are more invasive than other extra-abdominal lesions, leading to severe bone damage and skull base erosion, which are especially more common in children (9–11). EAF can affect the limbs and may lead to functional loss due to extensive resection, local recurrence, and radiation therapy (12). The growth rate of the lesion varies depending on the richness of the blood supply. In our case, we believe that the rich vascular system in the axilla enabled the tumor to grow to a size of 6 × 4 × 3 cm. Unfortunately, due to the rarity of this type of tumor, there are currently no clear diagnostic and treatment guidelines. Currently, the preferred examination methods include MRI and core needle biopsy. However, it should be noted that the final diagnosis requires a routine section examination of the surgically resected tissue. Other studies have reported that the location of DF affects the event-free survival (EFS) of patients after treatment (13, 14). In particular, lesions involving the abdominal wall, abdominal cavity, breast, digestive organs, and lower extremities seem to have a better prognosis. For these anatomical sites, the event-free survival after surgical treatment and active monitoring is similar. In contrast, for lesions in unfavorable locations, surgical treatment can significantly improve the EFS. However, due to the complex and important distribution of nerves and blood vessels in the axilla, this location is considered one of the “unfavorable locations” in our case.

More than 90% of cases of DF are sporadic, and trauma, gene mutations, genetic factors (Gardner’s syndrome), and endocrine disorders may be related to DF (15). Studies have suggested that somatic β-catenin-activating mutations and Wnt/β-catenin signaling pathway activation are thought to drive DF development (16, 17). Although the Wnt/β-catenin pathway is directly implicated in the pathogenesis of DF, the crosstalk with the Notch signaling pathway is critical, as druggable targets, like γ-secretase inhibitors (GSIs), are targeting the substrates in the Notch signaling pathway (18).

Imaging findings closely resemble those of invasive tumors. A typical B-ultrasound image shows low-echo nodules with irregular morphology and uneven edges, mostly presenting as non-parallel, angular spicules, and posterior echo attenuation (19). The above ultrasonic features are similar to those of cancer, which improves the imaging grading (BI-RADS) (20–22). Meanwhile, much literature reports that DF exhibits varying degrees of uniform enhancement in CT and MRI scans. In addition, they typically exhibit characteristics similar to malignant tumors in terms of signal intensity, tumor marginalization, and tendency to envelop adjacent organs (23). This is also the reason why the examination results of UT and MRI in the present study are more biased toward malignant tumors. However, MRI is considered a radiological tool for diagnosing, evaluating, and monitoring EAF. It can accurately determine the size, infiltration, and relationship with adjacent neurovascular structures of tumors. It is the best method for predicting the resectability of DF. Therefore, all patients should undergo baseline MRI examination before making any management decisions, except when there is the smallest potential disease of DF. The sensitivity of MRI in detecting disease progression, regression, or recurrence also makes it a preferred method during follow-up (24).

Pathological manifestations of DF are more uniformly moderate spindle fibroblasts or myofibroblasts arranged in a bundle, showing abundant collagen fibers with variable glass changes. The cells have no or mild atypia, nuclear spindle, and no necrosis. Lesions infiltrate fat or striated muscle tissue partially. Lymphocytic infiltration is often seen around the lesion, occasionally with lymphoid follicle formation. In the present study, lymphocyte infiltration was seen around the lesions, consistent with the literature (25–28). There is too little material obtained from fine-needle puncture to make a clear diagnosis, but it is worth noting that the appearance of myofibroblast proliferation or obvious collagenization in the stroma during fine-needle puncture can serve as diagnostic clues. Pathologists are reminded not to miss or misdiagnose it as glandular disease and should communicate with clinical physicians, radiologists, and patients to discuss further treatment methods. According to data, misdiagnosed cases in the initial examination of DF are approximately 30%–40% (29, 30). Furthermore, the misdiagnosis rate of fibromatosis is 10% to 28%. The initial examinations of UT and MRI in this study diagnosed this case as a malignant tumor. The results of immunohistochemistry showed that β-catenin, SMA, and desmin were positive, while CK, Estrogen Receptor (ER), Progesterone Receptor (PR), CK5/6, p63, CD34, and S-100 were all negative. β-Catenin nuclear positivity is an important biomarker for diagnosing invasive fibromatosis, but its specificity is not 100%. There is a report that the positivity rate of β-catenin is 78.9% (31), while some studies have found that the nuclear positivity rate of β-catenin is only 50%, and it is mostly individual or scattered in the nucleus (32).

Traditionally, surgical resection was the primary treatment adopted for DF. However, this tumor is recognized as highly unpredictable with high rates of local recurrenceand recurrence rates of up to 77% (33, 34), especially high within 3 years after the initial operation, which is mostly related to incomplete tumor resection orother high-risk factors for recurrence including young age and large tumor (35). A study found that the recurrence time of initial tumors ranged from 8 to 23 months with a median of 17.3 months, while the recurrence time of recurrent tumors ranged from 3 to 26 months, with a median of 14.8 months (36). Additionally, studies have shown that the average interval from primary to recurrence is 28 months (12). According to the European Guidelines, the “watchful waiting” strategy is the preferred therapy for asymptomatic DF (8). In addition, chemotherapy, non-steroidal anti-inflammatory drugs, endocrine therapy, and radiotherapy are also used in patients with tumor progression or inoperable tumors during follow-up (5). In the present study, although there was no relapse during the 2-year follow-up, regular follow-up examinations are still necessary.

**Table 1** summarizes the representative literature on breast fibromatosis in recent years.

**Table 1:** Summary of representative literature on breast fibromatosis (2017 –2025).

First author (year)	Age (years)/sex	Tumor location	Tumor size (cm)	Examination	Pre-treatment cytology/histology	IHC	Treatment	Follow-up
Amjad (2025) (37)	20/F	Left breast	1.5 × 1.5	UT, MG	Benign tissue with accompanying foci of adenosis and papilloma	Positive for smooth muscle actin, β-catenin, desmin, CD34, S-100, and CD10	Surgery	At 6 months, no recurrence
Zhao (2025) (38)	47/F	Left breast	6.6 × 2.2	UT, MRI, CT	DF	Not mentioned	Surgery	At 4 months, recurrence, palliative and supportive treatment
Sandoval (2024) (39)	28/M	Right breast	2.3	UT, MG, MRI	DF	β-Catenin positive	Surgery	At 2 years, no recurrence
Moussaddykine (2023) (40)	40/M	Upper quadrant of the left breast	4.1 × 3.9 × 3.7	MG, UT, MRI, CT	DF	Not mentioned	Surgery	Not mentioned
Laakom (2022) (41)	17/F	Left breast	4.1 × 1.6	MG, MRI	Fibrous remodeling, fragments of adipose tissue	Not mentioned	Surgery	At 3 years, no recurrence
	41/F	Right breast	2 × 1.5	UT	DF	β-Catenin positive	Surgery, radiotherapy	Not mentioned
Lin (2021) (19)	31/F	Upper outer quadrant of the left breast	2.0 × 1.5	MG, CEUS	DF	Positive for smooth muscle actin, desmin β-catenin, CD34, and Bcl-2	Surgery	At 6 months, recurrence, surgery
Boland (2021) (42)	21–70/F	Breast	0.9–6.0	UT, MRI	Bland spindle cell proliferation	Positive for actin, β-catenin, desmin S100 marker, and CD34	13 for surgery, 3 for surveillance	At 11 months 2 years, recurrence, “watch and wait” approach
Winkler (2021) (43)	38/M	Right breast	Not mentioned	MG, UT, MRI	DF	Positive for β-catenin	Surgery	At 6 weeks, recurrence, at 3 years, no recurrence
	48/F	Right breast	Not mentioned	CT, MG, MRI	Low-grade spindle cell neoplasm	Positive for β-catenin	Surgery	Not mentioned
Liu (2020) (44)	20–53/19F, 1M	Breast	0.2–11	MG, UT, MRI	DF	Not mentioned	Surgery	Not mentioned
Wuyts (2019) (22)	43/F	Left breast	0.7	MRI, MG, UT	DF	Not mentioned	Surgery	Not mentioned
Ghanta (2019) (45)	28–64/15F, 1M	Breast	1.5–15	MRI	Not mentioned	Positive for β-catenin, smooth muscle actin	Surgery, radiotherapy	2 had recurrence, mean follow‐up 65 months
Hill (2018) (46)	34/F	Left breast	11 × 8.0 × 4.5	UT, MRI	Spindle cell lesion, no cytologic atypia	Not mentioned	Surgery	At 8 months, no recurrence
Grimaldia (2017) (47)	31/F	Inferior outer quadrant of the left breast	2.3 × 1.0	UT, MRI	Proliferation of spindle cells, dense connective bundles	Positive for smooth muscle actin, β-catenin	Surgery	At 3 months, recurrence, surgery
Scheer (2017) (48)	19/F	Lower inner quadrant of the right breast	5.0 × 2.5	MG, UT, MRI	Proliferation of fibroblastic-like and myofibroblastic-like spindle cells	Negativity of anti-pan keratin antibodies, ER, protein S100, CD34, and calretinin	Initial medical treatment	At 13 months, tumor volume decrease of 57%
Kuba (2017) (49)	16–81/F	Breast	0.5–6.8	MRI	Spindle cell proliferation, DF	Positive for β-catenin	Surgery	At 0.7–220.8 months, no recurrence

IHC, immunohistochemistry; UT, ultrasonic testing; MG, mammography; DF, desmoid fibromatosis; CEUS, Contrast-Enhanced Ultrasound.

## 4 Conclusions

DF is a rare type of tumor, especially when it occurs in the accessory breast. It is usually invasive, is prone to recurrence after surgery, and has the potential risk of distant metastasis. Due to similar clinical and imaging manifestations, it may be misdiagnosed as a malignant tumor. Therefore, comprehensive clinical, imaging, and histological examinations are essential for making a diagnosis. There is still controversy regarding the optimal management of DF. A variety of treatment regimens have been reported, and wide surgical resection is the preferred method. Radiotherapy may be beneficial to postoperative patients by reducing the local recurrence rate. Up to now, due to the low incidence of this disease and limited data, there is still no standardized clinical treatment strategy. Moreover, because of its relatively high recurrence risk, further research is needed to more thoroughly determine the treatment plan and the recurrence risk. Our case can contribute to improving the understanding of this disease.

## Data Availability

The original contributions presented in the study are included in the article/supplementary material. Further inquiries can be directed to the corresponding author.

## References

[1] Kasper B Ströbel P Hohenberger P. Desmoid tumors: clinical features and treatment options for advanced disease. Oncologist. (2011) 16:682–93. doi: 10.1634/theoncologist.2010-0281 PMC3228186 21478276

[2] Fletcher CD Bridge JA Hogendoorn P Mertens F. WHO Classification of Tumours of Soft Tissue and Bone (IARC WHO Classification of Tumours). 4th ed. Lyon, France: IARC (2013).

[3] Lee YS Joo MW Shin SH Hong S Chung YG. Current treatment concepts for extra-abdominal desmoid-type fibromatosis: A narrative review. Cancers (Basel). (2024) 16:273. doi: 10.3390/cancers16020273 38254764 PMC10813957

[4] Garcia-Ortega DY Martín-Tellez KS Cuellar-Hubbe M Martínez-Said H Álvarez-Cano A Brener-Chaoul M. Desmoid-type fibromatosis. Cancers (Basel). (2020) 12:1851. doi: 10.3390/cancers12071851 32660036 PMC7408653

[5] Eastley N McCulloch T Esler C Hennig I Fairbairn J Gronchi A. Extra-abdominal desmoid fibromatosis: A review of management, current guidance and unanswered questions. Eur J Surg Oncol. (2016) 42:1071–83. doi: 10.1016/j.ejso.2016.02.012 26965303

[6] Wagstaff MJ Raurell A Perks AG. Multicentric extra-abdominal desmoid tumours. Br J Plast Surg. (2004) 57:362–5. doi: 10.1016/j.bjps.2004.02.014 15145742

[7] Bonvalot S Desai A Coppola S Le Péchoux C Terrier P Dômont J. The treatment of desmoid tumors: a stepwise clinical approach. Ann Oncol. (2012) 23 Suppl 10:x158–66. doi: 10.1093/annonc/mds298 22987953

[8] Wang J Huang Y Sun Y Ge Y Zhang M. Value of imaging findings in predicting post-operative recurrence of desmoid-type fibromatosis. Oncol Lett. (2020) 19:869–75. doi: 10.3892/ol.2019.11129 PMC6924159 31897201

[9] Murli D Smriti V Yadav S Trivedi B Qureshi SS. Desmoid fibromatosis of the oesophagus creating an oesophageal diverticulum in a 2-year-old girl. Afr J Paediatr Surg. (2024) 21:210–2. doi: 10.4103/ajps.ajps_120_22 PMC11379330 39162760

[10] Curry DE Al-Sayed AA Trites J Wheelock M Acott PD Midgen C. Oral losartan after limited mandibulectomy for treatment of desmoid-type fibromatosis. Ear Nose Throat J. (2023) 102:NP49–52. doi: 10.1177/0145561320987641 33491484

[11] Mohammadi S Mohammadi S Khosraviani F. A submandibular fibromatosis; A case report and review of literature. Med J Islam Repub Iran. (2022) 36:94. doi: 10.47176/mjiri.36.94 36419942 PMC9680815

[12] Asaad SK Abdullah AM Abdalrahman SA Fattah FH Tahir SH Omer CS. Extra-abdominal recurrent aggressive fibromatosis: A case series and a literature review. Mol Clin Oncol. (2023) 19:84. doi: 10.3892/mco.2023.2680 37808248 PMC10557105

[13] Penel N Le Cesne A Bonvalot S Giraud A Bompas E Rios M. Surgical versus non-surgical approach in primary desmoid-type fibromatosis patients: A nationwide prospective cohort from the French Sarcoma Group. Eur J Cancer. (2017) :83:125–131. doi: 10.1016/j.ejca.2017.06.017 28735069

[14] Duan M Xing H Wang K Niu C Jiang C Zhang L. A large and aggressive fibromatosis in the axilla: a rare case report and review of the literature. Onco Targets Ther. (2018) 11:3179–84. doi: 10.2147/OTT.S165209 PMC5983020 29881291

[15] Tzur R Silberstein E Krieger Y Shoham Y Rafaeli Y Bogdanov-Berezovsky A. Desmoid tumor and silicone breast implant surgery: is there really a connection? A literature review. Aesth Plast Surg. (2018) 42:59–63. doi: 10.1007/s00266-017-0948-2 28842766

[16] Mullen JT DeLaney TF Rosenberg AE Le L Iafrate AJ Kobayashi W. β-Catenin mutation status and outcomes in sporadic desmoid tumors. Oncologist. (2013) 18:1043–9. doi: 10.1634/theoncologist.2012-0449 PMC3780636 23960186

[17] Le Guellec S Soubeyran I Rochaix P Filleron T Neuville A Hostein I. CTNNB1 mutation analysis is a useful tool for the diagnosis of desmoid tumors: a study of 260 desmoid tumors and 191 potential morphologic mimics. Mod Pathol. (2012) 25:1551–8. doi: 10.1038/modpathol.2012.115 22766794

[18] Shang H Braggio D Lee YJ Al Sannaa GA Creighton CJ Bolshakov S. Targeting the Notch pathway: A potential therapeutic approach for desmoid tumors. Cancer. (2015) 121:4088–96. doi: 10.1002/cncr.29564 PMC4635059 26349011

[19] Lin S Cao Y Chen L Chen M Zhang S Jia X. Contrast-enhanced ultrasound of breast fibromatosis: a case report. J Int Med Res. (2021) 49:3000605211010619. doi: 10.1177/03000605211010619 33978517 PMC8120548

[20] Lou L Teng J Qi H Ban Y. Sonographic appearances of desmoid tumors. J Ultrasound Med. (2014) 33:1519–25. doi: 10.7863/ultra.33.8.1519 25063419

[21] Kumar A Kumar S Sinha R Kumari V. Largest size of extra-abdominal fibromatosis of axilla in a young man. BMJ Case Rep. (2019) 12:e230670. doi: 10.1136/bcr-2019-230670 PMC6677982 31375508

[22] Wuyts L De Schepper A. Desmoid-type fibromatosis of the breast mimicking carcinoma. J Belg Soc Radiol. (2019) 103:13. doi: 10.5334/jbsr.1612 30706051 PMC6354018

[23] Lee SB Oh SN Choi MH Rha SE Jung SE Byun JY. The imaging features of desmoid tumors: the usefulness of difusion weighted imaging to diferentiate between desmoid and Malignant soft tissue tumors. Investig Magn Reson Imaging. (2017) 21:162–70. doi: 10.13104/imri.2017.21.3.162

[24] Guglielmi G Cifaratti A Scalzo G Magarelli N. Imaging of superficial and deep fibromatosis. Radiol Med. (2009) 114:1292–307. doi: 10.1007/s11547-009-0458-7 19789958

[25] Munnangi A Kadapathri A Pillai V Bhat S Rajeswarie RT Shetty V. Isolated infratemporal fossa desmoid fibromatosis: A rare case report and review of literature. Indian J Otolaryngol Head Neck Surg. (2022) 74:2609–13. doi: 10.1007/s12070-020-02294-x PMC9702405 36452559

[26] Sato R Bandoh N Goto T Uemura A Inoue N Otomo Y. Resection of a desmoid-type fibromatosis with a CTNNB1 p.S45P mutation using a cervico-thoracic approach: A case report and literature review. Auris Nasus Larynx. (2021) 48:777–82. doi: 10.1016/j.anl.2020.05.004 32505607

[27] Remedios E Sommerfield D Fellingham W Powers N Stannage K Hii JWS. Paraspinal desmoid fibromatosis after lumbar epidural analgesia. Anaesth Rep. (2021) 9:e12129. doi: 10.1002/anr3.12129 34396135 PMC8340928

[28] Nisha S Chetana C Ranjini K Adarsh K. Desmoplastic fibroma of the Mandible with unusual histopathological features. Indian J Pathol Microbiol. (2021) 64:548–52. doi: 10.4103/IJPM.IJPM_698_20 34341270

[29] Kasper B Baumgarten C Bonvalot S Haas R Haller F Hohenberger P. Management of sporadic desmoid-type fibromatosis: a European consensus approach based on patients’ and professionals’ expertise-a sarcoma patients EuroNet and European Organisation for Research and Treatment of Cancer/Soft Tissue and Bone Sarcoma Group initiative. Eur J Cancer. (2015) 51:127–36. doi: 10.1016/j.ejca.2014.11.005 25434922

[30] Penel N Coindre JM Bonvalot S Italiano A Neuville A Le Cesne A. Management of desmoid tumours: A nationwide survey of labelled reference centre networks in France. Eur J Cancer. (2016) 58:90–6. doi: 10.1016/j.ejca.2016.02.008 26974708

[31] Norkowski E Masliah-Planchon J Le Guellec S Trassard M Courrèges JB Charron-Barra C. Lower rate of CTNNB1 mutations and higher rate of APC mutations in desmoid fibromatosis of the breast: A series of 134 tumors. Am J Surg Pathol. (2020) 44:1266–73. doi: 10.1097/PAS.0000000000001517 32590455

[32] Tu J Yan H Fu L Zhang H Wang Y Zhong S. Breast fibromatosis:a clinicopathologic analysis of fourteen cases and review of literature. J Diag Pathol April. (2024) 31:318–22. doi: 10.3969/j.issn.1007-8096.2024.04.010

[33] Sedaghat S Sedaghat M Krohn S Jansen O Freund K Streitbürger A. Long-term diagnostic value of MRI in detecting recurrent aggressive fibromatosis at two multidisciplinary sarcoma centers. Eur J Radiol. (2021) 134:109406. doi: 10.1016/j.ejrad.2020.109406 33254066

[34] Nuyttens JJ Rust PF Thomas CR Jr Turrisi AT 3rd. Surgery versus radiation therapy for patients with aggressive fibromatosis or desmoid tumors: A comparative review of 22 articles. Cancer. (2000) 88:1517–23. doi: 10.1002/(SICI)1097-0142(20000401)88:7<1517::AID-CNCR3>3.0.CO;2-9 10738207

[35] Lorenzen J Cramer M Buck N Friedrichs K Graubner K Lühr CS. Desmoid type fibromatosis of the breast: ten-year institutional results of imaging, histopathology, and surgery. Breast Care (Basel). (2021) 16:77–84. doi: 10.1159/000507842 33708054 PMC7923936

[36] Wang YF Guo W Sun KK Yang RL Tang XD Ji T. Postoperative recurrence of desmoid tumors: clinical and pathological perspectives. World J Surg Oncol. (2015) 13:26. doi: 10.1186/s12957-015-0450-8 25888954 PMC4329213

[37] Amjad A Shaikh K Idress R Zeeshan S. Desmoid fibromatosis of the breast. BMJ Case Rep. (2025) 18:e264208. doi: 10.1136/bcr-2024-264208 40132919

[38] Zhao Z Shang Q Yang C Liu J Liu S Li X. Desmoid-type fibromatosis of the breast: a case report and literature review. Front Oncol. (2025) 15:1482024. doi: 10.3389/fonc.2025.1482024 40008005 PMC11850368

[39] Correa Sandoval DC Gonzalez Reyes J Guajardo Nieto DA Guzman Murguia JL. Desmoid-type fibromatosis of the breast in a male patient following cosmetic surgery: a rare case report. Front Oncol. (2024) 14:1438050. doi: 10.3389/fonc.2024.1438050 39741980 PMC11685193

[40] Moussaddykine S Sy N’deye M. Desmoid fibromatosis in a male breast with gynecomastia: A case report. Radiol Case Rep. (2023) 19:107–10. doi: 10.1016/j.radcr.2023.09.104 PMC10630553 38028308

[41] Laakom O Bergaoui H Hammouda SB Khalfalli A Njim L Faleh R. Fibromatose desmoïde mammaire: à propos de deux cas et revue de la literature [Desmoid-type fibromatosis of the breast: about two cases and literature review. Pan Afr Med J. (2022) 41:184. doi: 10.11604/pamj.2022.41.184.28549 35655675 PMC9120741

[42] Boland MR Nugent T Nolan J O'Mahony J O'Keeffe S Gillham CC. Fibromatosis of the breast: a 10-year multi-institutional experience and review of the literature. Breast Cancer. (2021) 28:168–74. doi: 10.1007/s12282-020-01145-5 32780320

[43] Winkler N Peterson M Factor R. Breast fibromatosis: radiologic-pathologic correlation. J Breast Imag. (2021) 3:597–602. doi: 10.1093/jbi/wbab051 38424943

[44] Liu H Zeng H Zhang H Wang H Cheng Z Hu Y. Breast fibromatosis: Imaging and clinical findings. Breast J. (2020) 26:2217–22. doi: 10.1111/tbj.14008 32754995

[45] Ghanta S Allen A Vinyard AH Berger R Aoun J Rosenkrantz Spoont J. Breast fibromatosis: Making the case for primary vs secondary subtypes. Breast J. (2020) 26:697–701. doi: 10.1111/tbj.13506 31429159

[46] Hill E Merrill A Korourian S Bryant-Smith G Henry-Tillman R Ochoa D. Silicone breast implant associated fibromatosis. J Surg Case Rep. (2018) 2018:rjy249. doi: 10.1093/jscr/rjy249 30279974 PMC6158700

[47] Grimaldi MC Trentin C Lo Gullo R Cassano E. Fibromatosis of the breast mimicking cancer: A case report. Radiol Case Rep. (2017) 13:1–5. doi: 10.1016/j.radcr.2017.09.011 29487630 PMC5826686

[48] Scheer L Lodi M Molière S Kurtz JE Mathelin C. Medical treatment of mammary desmoid-type fibromatosis: which benefit? World J Surg Oncol. (2017) 15:86. doi: 10.1186/s12957-017-1148-x 28420393 PMC5395853

[49] Kuba MG Lester SC Giess CS Bertagnolli MM Wieczorek TJ Brock JE. Fibromatosis of the breast: diagnostic accuracy of core needle biopsy. Am J Clin Pathol. (2017) 148:243–50. doi: 10.1093/ajcp/aqx065 28821190

